# Differences and Relationship between Body Composition and Motor Coordination in Children Aged 6–7 Years

**DOI:** 10.3390/sports12060142

**Published:** 2024-05-23

**Authors:** Vladan Pelemiš, Slobodan Pavlović, Danimir Mandić, Milan Radaković, Dragan Branković, Vladimir Živanović, Zoran Milić, Senad Bajrić

**Affiliations:** 1 Faculty of Education, University of Belgrade, 11000 Belgrade, Serbia; dekan@uf.bg.ac.rs (D.M.); dragan.brankovic@uf.bg.ac.rs (D.B.); vladimir.zivanovic@uf.bg.ac.rs (V.Ž.); 2 Faculty of Education in Užice, University of Kragujevac, 34000 Kragujevac, Serbia; slobodan.b.pavlovic@gmail.com; 3 Faculty of Sport, University “Union—Nikola Tesla’’, 11000 Belgrade, Serbia; radakovic.milan@fzs.edu.rs; 4 College for Vocational Education of Preschool Teachers and Sport Coaches, University of Novi Sad, 24000 Subotica, Serbia; zoranmilic@yahoo.com; 5 Faculty of Sport Science, Pan-European University Apeiron, 78000 Banja Luka, Bosnia and Herzegovina; senad.o.bajric@apeiron-edu.eu

**Keywords:** KTK test, motor skills, relations, preschool age

## Abstract

Background: The primary goal of this study was to investigate the relationship between body composition and motor coordination performance, and the secondary goal was to determine sex differences in body composition and motor coordination of preschool children. Methods: Forty-eight children (23 boys and 25 girls) underwent assessments for body composition and motor coordination using the Köperkoordinationstest für Kinder (KTK). Results: Linear regression analysis revealed significant associations between body composition and motor coordination in boys (*p* < 0.05) but not in girls. In boys, Body height (*p* = 0.01), Total muscle mass (*p* = 0.03), Total fat (*p* = 0.03), and Total water (*p* = 0.02) show statistically significant influence on single-leg jumps. Similar results were obtained for lateral jumps where there was a statistically significant influence of Body height (*p* = 0.01), Total muscle mass (*p* = 0.03), and Total water (*p* = 0.02). Interestingly, predictive variables showed no statistically significant influence on KTK overall score in boys (*p* = 0.42) nor in girls (*p* = 0.90). Conclusions: The predictive system of morphological variables demonstrated significance only among boys in this age group and sample. Girls outperformed boys due to early maturation, resulting in better average KTK scores.

## 1. Introduction

Regular physical activity during childhood has been associated with reduced adiposity levels, enhanced cognitive skills, improved psycho-social characteristics, and better cardiovascular function [1]. Notably, the cornerstone of physical activity during the preschool years lies in the mastery of movement, which involves intricate interactions between the environment and the neuro-muscular system, constituting motor coordination [2]. 

Preschool institutions are presumed to offer an ideal setting for structured physical activities and overall motor skill development due to the extensive time children spend within them [3,4]. However, one study indicated that the time spent in preschool institutions tends to be sedentary in nature, potentially negatively impacting children’s overall motor behavior [5]. Additionally, sex differences emerge as significant factors influencing physical activity levels in preschool institutions, with boys exhibiting higher activity levels compared to girls [6]. 

Body composition dominated by adipose tissue poses a significant threat to the normal functioning of children and young individuals, impairing their movement abilities, reducing coordination levels, limiting participation in games and physical activities with peers, and negatively impacting psycho-social well-being [7]. Notably, body composition undergoes changes alongside the growth and development of children’s organisms, influenced by a variety of exogenous factors, such as environment and diet, as well as endogenous factors, including heredity [8]. Both body composition and regular physical activity serve as crucial predictors significantly influencing children’s coordination and performance in assessments like the Körperkoordinationstest für Kinder (KTK) [9]). The most recent study [10] examined the reliability of KTK tests and confirmed that the KTK is a reliable instrument for assessing the motor coordination of children. Studies have highlighted an inverse relationship between body mass and motor coordination [11,12]. While these studies primarily focused on body mass index, it is imperative to delve into the intricacies of body composition for a more comprehensive understanding of its impact on coordination development, as elucidated in this research. For instance, D’Hondt et al. [13] demonstrated in a cohort of Belgian children that excessive weight, particularly obesity, led to poorer performances in KTK tests, notably affecting dynamic body coordination. Similar findings were observed in studies involving Portuguese children by [14] Antunes et al., where normal-weight children outperformed their obese peers. Conversely, Aivazidis et al. [15] underscored the critical role of guided physical activity in preschool institutions for maintaining normal functioning and enhancing performance in KTK tests among children. Webster et al. [16] investigated the relationship between fundamental motor skills (locomotor and object control skills) and body composition (fat mass and percent, as well as fat-free mass) in 3–10-year-old children. They found that the 23% variance in locomotor skills (i.e., run, gallop, hop, leap, jump, and slide) may be explained by the body composition measures, although only fat-free mass contributed significantly to the regression model.

Childhood obesity is a significant public health issue globally. Growing recognition of the problem paralleled escalating rates of childhood obesity and evidence establishing the links with a number of serious metabolic, physical, social, and psychological consequences [17]. The World Health Organization [18] published a report stating that some countries reported more than 40% of children living with obesity, especially in Greece, Italy, Portugal, and Spain, and that despite a reduction of 5–10%, the prevalence of overweight and obesity remained high in these countries. A similar reduction was observed in Slovenia, where the initial value of overweight prevalence was significantly lower (about 30%). According to the same study, data from Serbia for children aged 7–9 show that 15% were obese, and around 33% were overweight. It is also significantly less than the 2013 report published by the Institute for Public Health of Serbia “Dr. Milan Jovanović Batut” [19].

Recent research conducted by Italian scientists [20] has revealed a concerning trend indicating a decline in the motor quotient observed in Körperkoordinationstest für Kinder (KTK) assessments. This downward trajectory suggests that contemporary youth are acquiring fewer significant motor experiences compared to their counterparts in the 1970s [21]. Supporting this notion, Italian data showed lower scores than German references and Belgian results but slightly higher scores than Brazilian results within the same sample of participants [20]. A comparative analysis of these findings highlights the alarming trend of diminishing overall motor proficiency and its variation across different geographical and socio-cultural contexts. 

This study should provide clearer knowledge about which segments of body composition contribute the most to the association of performance of motor coordination in the oldest group of preschool children and identify the possibility of sex differences. There is a lack of comprehensive investigation regarding the relationship between body composition and motor coordination specifically tailored to preschool-aged children, as well as the scarcity of studies examining potential sex differences in these associations within this demographic. Therefore, the primary goal of the study would be to determine the connection between body composition and motor coordination performance, and the secondary goal would be to determine the sex differences of preschool children in body composition and motor coordination. The aim of this study was to investigate the difference in motor coordination between boys and girls of preschool age, as well as to establish the influence of body composition on motor coordination. These tests are renowned for their reliability and standardization, providing a balanced assessment of motor skills aligned with the growth and development of children.

## 2. Materials and Methods

The conducted study was empirical cross-sectional research. Data collection involved the utilization of standardized on-field tests for assessing motor skills, alongside a validated instrument for estimating body composition among preschool-aged children. Subsequently, various statistical methods were employed for data analysis, and the obtained results were interpreted using bibliographical and speculative approaches.

### 2.1. Participants

The sample of respondents for the purposes of the research was derived from the population of children of preschool age using the non-probability sampling method, i.e., quota sample. This type of sample has a professional and scientific justification in cases where mass phenomena are investigated and groups (samples) were previously homogenized [22]. All participants were free of musculoskeletal, neurological, and orthopedic disorders. Additionally, all of the participants were free from diabetes, congenital disorders, or any other metabolic syndrome conditions. Except for the regular physical education curriculum in kindergarten, the participants were not involved in any additional form of physical exercise outside their institution. 

The sample comprised a total of 48 participants, with an average age of 6.52 ± 0.34 years, including 23 boys of the oldest preschool groups (average age 6.58 ± 0.35 years) and 25 girls of the oldest preschool groups (average age 6.46 ± 0.32 years) from the “Užice” preschool institution located in Užice. The total number of enrolled children of the oldest preschool groups in the preschool institution was 314 in 11 kindergartens in the territory of the city of Užice. The selected sample represented about 15% of the total number of enrolled children in the oldest preschool groups. Motor skill assessments and body composition evaluations were conducted at the outset of October 2023. Upon obtaining informed consent from parents/guardians, the measurement and evaluation of participants’ characteristics and abilities commenced. It is noteworthy that all subjects were healthy individuals devoid of any mental or physical impairments.

Ethical clearance for the study was granted by the Faculty of Education in Užice, University of Kragujevac (Reg. No.: 28/092023, approval date: 2 September 2023), and all experiments were conducted in strict accordance with the principles outlined in the Declaration of Helsinki.

### 2.2. Testing

Among all morphological characteristics considered as predicting variables in this research, the following were taken into account: Body height (cm) for estimating dimensions and body length; Body mass (kg) for estimating voluminosity and overall body mass; Total muscle mass (0.1 kg), Total fat (0.1 kg), and Total water (0.1 kg) for assessing body composition.

The Körperkoordinationstest für Kinder (KTK) test, a highly dependable standardized motor skills assessment tool, was utilized to evaluate motor coordination [23]. This test is designed for children over the age of five and provides normative data. It comprises four subtests: balance beam, single-leg jumps, lateral jumps, and platform movement.

To determine a child’s coordination level, the total raw values obtained from individual attempts in each KTK subtest were summed. However, raw values alone do not indicate a child’s level of performance; they must be compared to average values within a specific age group.

Based on the overall Motor Quotient (MQ) value, a child’s coordination level can be classified as follows:

Very low (obstructed body coordination): MQ ≤ 70; ≤2nd percentile

Low (unusual body coordination): MQ = 71–85; 3rd–16th percentile

Normal (typical body coordination): MQ = 86–115; 17th–84th percentile

High (good body coordination): MQ = 116–130; 85th–98th percentile

Extremely high (excellent body coordination): MQ ≥ 131; ≥99th percentile.

#### 2.2.1. Balance Beam

Time of action: about 3–4 min per examinee. Number of examiners: one examiner, one assistant. Props: 3 beams 3 m long, 6 cm, 4.5 cm, and 3 cm wide. Process description: in a gym with a flat surface, without any slopes, on the surface of 5 m × 5 m, set the beams in a parallel position by first setting the 6 cm wide beam (yellow); then, next to it, the 4.5 cm wide beam (red), and the last 3 cm wide beam (green). Task: As a trial attempt, the examinee should first walk along the entire 6 cm beam forward. After that, as a task that is measured, he/she has 3 attempts to walk along the beam backward. He/she should repeat the task with the 4.5 and then 3 cm wide beams. The performance of the task: by walking backward, the examinee should keep the balance and successfully make a maximum of 8 steps backward for each of the 3 attempts. The end of the task performance: the task ends for each of the 3 attempts when the examinee loses balance and, with one foot, touches the ground below the beam. The examiner’s position: the examiner follows the examinee all the time and notes the attempts or tells them to the assistant who records them. Assessment: the score is the sum of all 9 attempts on 3 beams. Since the maximum number of steps is 8, the total score cannot be higher than 72.

#### 2.2.2. Single-Leg Jumps

Time of action: about 4–5 min per examinee. Number of examiners: one examiner, one assistant. Props: 3 mats with the following dimensions: 2 m × 1 m, 12 sponges with the following dimensions: 60 cm × 20 cm × 5 cm. Process description: in a gym with a flat surface, without any slopes, on the surface of 8 m × 4 m, set the mats lengthwise, next to each other. On the junction between the second and the third mat, sponges should be set. Task: As a trial attempt, the examinee should first jump over one sponge using one leg, first left and then right. After the run using one leg, the examinee should jump over the 0–60 cm high obstacle. The performance of the task: after the run using one leg, the examinee should jump over the obstacle and land on the same leg that he used as a take-off, after which he is due to jump on that very same leg at least 2 more times. The end of the task performance: the task ends when the examinee makes a mistake 3 times (knocks down the sponge, does not continue jumping on the leg that he bounced off after jumping over the obstacle). The examiner’s position: the examiner stands opposite the sponge and notes the scores or tells his assistant to record them. Assessment: the score is the sum of all successful attempts with both the left and right leg. If the obstacle is jumped over during the first attempt, the score is 3 points; from the second attempt, 2 points; and from the third attempt, 1 point. The maximum score is 78 points. Note: the task is performed alternately once and then with the other leg for the obstacle of the same height. 

#### 2.2.3. Lateral Jumps

Time of action: about 1 min per examinee. Number of examiners: one examiner. Props: 1 crossbar with the following dimensions: 60 cm × 4 cm × 2 cm and a stopwatch. Process description: jump over the crossbar with a side two-foot jump as many times as possible during 15 s. Do the test twice. Task: As a trial attempt, the examinee should perform 5 jumps. The performance of the task: the examiner gives the signal that the examinee can start the test and, using the stopwatch, measures 15 s. Moreover, in those 15 s, the examiner counts the successful jumps. The end of the task performance: the tasks end after 15 s. The examiner’s position: the examiner stands in front of the examinee and notes the score. Assessment: the score is a sum of all successful jumps, both for the first and the second attempt. 

#### 2.2.4. Platform Movement

Time of action: about 1 min per examinee. Number of examiners: one examiner. Props: 2 wooden platforms with the following dimensions: 25 cm × 25 cm × 5.7 cm and a stopwatch. Process description: 2 platforms are set next to each other, and the examinee stands on the right one if the direction of movement is on that side or on the left one if the direction of movement is to the left side. Move the platform from one side to the other in the direction of movement and then stand on it. Repeat the task as many times as possible in 20 s. Perform the test twice. Task: As a trial attempt, the examinee should perform 5 movements. The performance of the task: the examiner gives the signal that the examinee can start the test and, using the stopwatch, measures 20 s. Moreover, in those 20 s, the examiner notes down the number of platform movements. The end of the task performance: the tasks end after 20 s. The examiner’s position: the examiner stands in front of the examinee and notes down the number of movements. Assessment: the score is a sum of all movements, both for the first and the second attempt.

Body height was measured in a standing position using an anthropometer according to Martin (Sieber Hegner Maschinen AG, Zürich, Switzerland) with an accuracy range of 0.01 mm.

Body composition variables were measured with the In-Body 230 (Biospace Co., Seoul, Republic of Korea) using the direct segmental multi-frequency bioelectrical impedance analysis (DSM–BIA) method and included 4 outcome measures: body mass, total muscle mass, total fat mass, total body water. Height measurements, age, and sex were manually inserted into the BIA device. Prior to testing, the subjects were instructed not to eat anything in the morning, to avoid any kind of exercise 24 h before analysis, and to perform all physiological needs before the measurement. Subjects were in the standing position for at least 5 min prior to the measurement for the redistribution of body fluids. During the measurement, all subjects were in light sports clothing and had no metal accessories. There were no predetermining parameters to control/normalize all subjects [24].

### 2.3. Data Analysis

Statistically–mathematical data processing included the calculation of descriptive statistics, arithmetic mean, and standard deviation. For the estimation of distribution normality, the Shapiro–Wilk coefficient on the reference level of *p* ≤ 0.05 was used here. The existing statistically significant difference between the 2 subsamples for all analyzed variables will be tested with the help of a *t*-test for independent samples (*p* ≤ 0.05). With the help of linear regression analysis, the influence of the set of predictive (morphological) variables on the motor variables for estimation of coordination has been established, and those variables presented the criteria variables in the work.

## 3. Results

The values of the t-test for the independent sample (Table 1) pointed to the fact that there was no significant difference between boys and girls in body composition (*p* < 0.05).

The values of descriptive statistics for motor coordination (Table 2) point to the better average results in favor of girls in single-leg jumps, where there is a significant statistical difference (*p* = 0.01). 

Further, we have presented the regression analysis showing the influence of body composition on motor coordination (Table 3, Table 4, Table 5, Table 6 and Table 7). The regression analysis with subsamples of boys and girls (Table 3) showed there was no significant influence of the system of predictive variables on the examined criterion (boys P = 0.86 and girls P = 0.97), with the value for the coefficient of multiple correlation R = 0.32 and R = 0.20, respectively. Accordingly, none of the predictive variables showed statistically significant influence on the examined criterion, both with boys and girls (*p* < 0.05).

Using regressive analysis of criterion variable single-leg jumps (Table 4) with boys, it was established that there is a statistically significant influence of the system of predictive variables on the examined criterion because the significance of the multiple correlation coefficient P = 0.00, and the value of multiple correlation coefficient R = 0.79. Such connection is explained by 62% of the mutual variability of the predictive system and the criterion. 

Predictive variables, Body height (pbeta = 0.00), Total muscle mass (pbeta = 0.03), Total fat (pbeta = 0.03), and Total water (pbeta = 0.02), show statistically significant influence on reflecting this motor task. There was no statistically significant correlation of all predictive variables with the criterion. The biggest (positive) influence was acquired by Total water variable (Beta = 5.36).

With the subsample of girls examinees, there was no statistically significant influence of the system of predictive variables on the same criterion because the significance of the multiple correlation coefficient P = 0.97, that is, the value of multiple correlation coefficient R = 0.25, whereas the mutual described variability was only 6%. Some other characteristics and abilities have a greater impact on the reflection of entire body coordination with girls.

Reviewing the results of the regressive analysis of the lateral jumps variable (Table 5), it can be noticed that there is a statistically significant influence of the system of predictive variables on the criterion variable with the subsample of boys (P = 0.02) with the value R = 0.73, which describes 53% of mutual variability, whereas with girls there is no statistical significance (P = 0.70). Relatively low values of multiple correlation coefficient R = 0.37 explain only 14% of mutual variability with boys. There is a statistically significant influence of the Body height variable (pbeta = 0.00), Total muscle mass (pbeta = 0.03), and Total water (pbeta = 0.02) on the reflection of this motor task with boys. Taller boys with higher values of muscle mass and a lower percentage of water achieved lower scores. The biggest (positive) influence is the Total water variable. 

There were no statistically significant correlations between the predictors and the criterion variable with boys and girls.

Reviewing the results of the regressive analysis of the platform movement variable, it can be noticed that there is a statistically significant influence of the system of predictive variables on the criterion variable with boys (P = 0.22) and with girls (P = 0.26). The value of the multiple correlation coefficient R = 0.56 with boys and R = 0.52 with girls explains only 22% with boys and 26% of mutual variability with girls. Only the Total fat variable with boys showed a statistically negative influence on performing this motor task (pbeta = 0.03), but since the system was not statistically significant, this can be regarded as accidental, considering that there was no statistically significant correlation of the same predictor and the criterion with this group of examinees.

The results of the regressive analysis of the KTK overall score variable (Table 7) point out that there was no statistically significant influence of the system of predictive variables on the criterion variable with boys (P = 0.42) nor with girls (P = 0.90). The value of the multiple correlation coefficient R = 0.49 with boys and R = 0.28 with girls explains only 26% with boys and 8% of the mutual variability with girls. 

It is necessary to point out that none of the predictive variables pointed to some statistically significant influence on the criterion (pbeta < 0.05) with both groups.

## 4. Discussion

The primary aim of the study was to investigate the association between coordination and components of body composition. The scientific literature contains reports on multiple tests used to analyze patterns for monitoring children’s motor skills. However, some variables can influence the results of certain tasks that are part of these motor coordination tests. This is the case for the influence of body composition in the Korperkoordinationstest fur Kinder (KTK) [14,20], test battery. There is also a connection between the psychological component and performance in KTK tests. Namely, the influence of family motivation on greater gains in motor performance has been proven [25]. 

The results of this research pointed to the consistent body growth and motor development of boys and girls. However, boys were, on average, taller and weighed more than girls, and they had a higher percentage of muscle and a lower percentage of body fat. Comparing our results to the results of Haapala et al.’s [26] research, it can be noticed that the boys and girls from Serbia had lower percentages of Total muscle mass compared to the children from Finland (13.0% to 11.43% girls and 15.5% to 12.5% with boys). The boys from Finland had higher values of Total fat compared to the boys in the current research (6.8% to 5.89%), whereas the girls had lower values (4.2% to 6.86%). Obviously, there are some socio-cultural differences that influenced such results. Nonetheless, the secondary aim of the study leads to the conclusion that girls dominate regarding only one variable, single-leg jumps, compared to the boys of a similar age and that the system of predictive variables had a statistically significant influence on the reflection of results in two variables of the KTK test namely only with boys (single-leg jumps and lateral jumps). Our results are contrary to the results of Fransen et al. [27] and Bardid et al. [28], which point out that there is no significant difference between sex with children of 6–7 years of age. Girls in the current research had slightly higher levels of motor coordination compared to boys, which can be justified by the earlier development (growing up) and not the sexual maturity of girls at this age. Higher values of motor coordination tests in girls show a higher level of maturity compared to boys at this age of growing up. This is not changing data, considering that girls are about 6 months more mature than boys at this age. Specifically, the greatest difference in motor coordination tests was found in the variable single-leg jumps (10.68 to 7.48) in favor of girls. The earlier development of girls could be seen through the higher percentage of Total fat in girls because their cells are filling with adipose tissue before the mere division of cells [2]. Maybe the differences in the variable of single-leg jumps can be justified by everyday activities because the movements of this structure are a part of many activities in most daily activities of children, so this could be the reason for the great difference between girls and boys, but also lower average values of body mass with girls. Still, research showed that the maturing aspect presents a negative correlation with motor performances, mostly related to the movement backward (balance beam), in which children who have slightly deterred biological maturing show better performance. However, this was not the case in our results because, on this test, girls had better average results than boys. These differences are similar to earlier results in studies by Rocha and Fernandes [29], as well as in D’Hondt et al., [12]. Some studies [30,31], however, point out that growing up was an insignificant factor that influenced the motor test, with the note that the growing up processes are not the only aspects which are in direct connection to better performance in tasks which estimate motor coordination on the KTK test. Our results further showed that certain parameters of body composition (Body height, Body mass, Total muscle mass, Total fat, and Total water) had an influence on motor coordination, confirming the results of Hardman [9], who showed that variables of body composition can affect the results on the KTK test. Since our study showed significant influences of Total muscle mass on the results of KTK, it can be pointed out that it is one of the predictors of motor coordination in the period of childhood, which was also shown in some previous research [32]. Studies have constantly shown similar results in the last 20 years that higher body mass in children is associated with a lower level of motor coordination [9,12,32,33,34], which was confirmed in our research as well, especially in boys. The results of regression analysis pointed out that Total muscle mass at this age has a negative influence on motor coordination in boys (which matches the research of Chagas & Batista [35], who point out that apart from BMI, body mass and the percentage of fat tissue had a great influence on motor skills. The current results showed that there is no association of body composition with the results of the lateral jumps and one-leg jumps test, while the higher water level showed an association with two motor tasks: one-leg jumps and lateral jumps. 

We can assume that the level of physical activity of those children and time spent in non-structured playing should be taken into account. It can also be assumed that some other characteristics and abilities at this age play a significant role in the development of motor coordination, which can be justified by the fact that girls showed no significant association between the predictive system and motor coordination and that there was a significantly low level of mutual variability in the range of 4 to 28%. On the contrary, the linear regression pointed to the fact that the chosen predictive system predicts motor coordination in boys with a relatively good percentage of mutual variability from 53 to 62%. Therefore, children’s body composition significantly contributes to the level of motor coordination. This is an important finding, especially when you take into account the reported individual as well as sex differences in KTK performances. The factor of physical activity was not taken into consideration in our research, but it can be assumed that it will most likely influence motor coordination [36]. Our findings indicate a negative link between total fat and motor coordination, particularly among boys. Consequently, we might anticipate even poorer outcomes among boys with higher fat levels, impacting their body mass, unless there are changes in their growth, development, or unhealthy habits. Probably, bad life habits are responsible for the condition those children are now in, and they score much lower results than the boys and girls who have lower levels of fat and body mass. 

It is well documented that the motor performance of children is usually influenced by the physical aspects of the examined individuals, such as height and percentage of body fat [14,37]. Body composition is a predictor that has a direct influence on motor performance on KTK tests regardless of the age and sex of an individual [13]. Our research results are confirmed by the research results of [38,39,40], where some negative correlation and influences of excessive body mass were established when solving the motor performances of the KTK test. This was also confirmed by the most recent research results [41]. The greatest negative correlation in the named studies was body mass, whereas in our research, Total water is pointed as a positive predictor and Total muscle mass as a negative predictor whereas the similarity in results of the named studies can be found in the negative influences of Body mass. The results of D’Hondt et al. [13] also pointed to the negative aspects of body mass and sub-skin fat, which can be connected to total fat, and the results of the KTK test. This group of authors also points out that with children, the voluminosity of the body, Body mass, and indirectly, BMI, are significant predictors of KTK performance, explaining it with 37.6% of its variance, which is lower than in our research, where body composition showed the level of variability from 53 to 62%. With more parameters, the values of mutual variability became clearer. Unlike the research of D’Hondt, et al. [13], the evolution of the level of gross motor coordination (Total KTK) was not significantly connected with body mass, although there was a negative influence on the total score. Moreover, Lopes et al. [32] documented the existence of the influence of sub-skin fat on the results of KTK. According to that, it seems that a child’s weight condition negatively influences motor coordination and vice versa. Unlike the research of Ré et al. [42], where boys had on average better values than girls in the results of the total KTK test in preschool age (102.8 to 98.3 points), in our research, girls had on average better results (89.56 to 85.91 points). Furthermore, the results of this sample of children are even lower than the results of Ré et al.’s [42] research, which points to the fall in motor abilities of this group of children. It could be due to lower physical activity, the way of life, and a sedentary lifestyle. 

In the variable of the Motor quotient, there were no stated statistically significant differences between boys and girls within the given sample of participants. The biggest percentage of participants had normal coordination (point 3), but there were no greater differences when it came to low or high coordination. Therefore, it can be assumed that in this period of childhood, different characteristics and abilities can influence the results of the KTK test. Previous studies that used this test confirmed that factors such as the stage of development [43], environment [44], and body composition [39] influence the results (both in positive and negative context) in the motor quotient of the KTK test with children who are active as well [30]. In that sense, it is reasonable to conclude that countless numbers of extrinsic factors can influence the results of motor tests such as KTK, but it is still not clear in the scientific literature which factors have the biggest influence on KTK results. Maybe, because of that, there were no significant differences in the Motor quotient with children of preschool age in our research. The World Health Organization mainly focuses on the recommendation in later periods of preadolescence or adolescence when some greater changes in body structure in children occur. 

Contextual factors, such as local and regional traditions, are often an obstacle to establishing a general overview of the factual situation in the territory of the entire country. Because of the distinct ground structure and the variability of samples in the Republic of Serbia, it would be prudent to explore different regions within Serbia and consider conducting a comparative analysis. Also, there is a need for the development and application of a scale that measures the integrated regulation of children for physical activities in order to obtain a more complete picture of student motivation in physical education activities. The limitation of the study with regard to the sample size should also be noted, which threatens the external validity of the research. The conclusions could not be applied to the whole country’s population, only to one specific part. Additionally, this study is cross-sectional, so the results obtained from a future longitudinal study would be of great importance for further discussion and proposed solutions. It is recommended that this and similar research be performed at other ages, especially in the early adolescent period.

## 5. Conclusions

The system of predictive variables showed a statistically significant association between the two criteria variables, single-leg jumps and lateral jumps, namely only with boys. It was established that girls have higher levels of motor coordination. Based on the results of this study, it can be assumed that the results will be of used in the development and monitoring of motor coordination of children 6–7 years of age from Serbia. Therefore, it should take into consideration introducing long-term and multisport programs of activity and encourage parents, teachers, and other participants in upbringing and education into the active realization of curriculum and extra curriculum time. An interesting topic for future research is to thoroughly examine the relationship between possible changes in body mass and total muscle mass and total fat, which will surely change throughout the period of growing up, with the coordination performances using the same battery of tests, especially in the period of adolescence and preadolescence.

## 6. Practical Applications

The analyzed results of the research will contribute to the eventual improvement of optimal physical activity programs for preschool children before entering primary school, which should be based on coordination content, as well as the possibility of planning an increase in the level of physical activity in order to improve the motor quotient for both sexes. The results have practical implications in the field of public health, as well as for actions aimed at increasing motor skills, monitoring the morphological growth and development of children after the age of seven, and constantly controlling obesity, which is a significant health problem in the Republic of Serbia.

## Figures and Tables

**Table 1:** Descriptive statistics and differences in body composition.

Variables	Boys (N = 23)		Girls (N = 25)				
	Mean ± SD	SWp	Mean ± SD	SWp	*t*	*p*	df
Body height (cm)	130.63 ± 5.59	0.40	128.86 ± 7.56	0.50	0.92	0.36	46
Body mass (kg)	28.02 ± 5.08	0.02	26.88 ± 5.90	0.04	0.72	0.48	46
Total muscle mass (0.1 kg)	12.50 ± 2.05	0.24	11.43 ± 2.25	0.02	1.71	0.09	46
Total fat (0.1 kg)	5.89 ± 3.16	0.00	6.86 ± 4.33	0.00	−0.89	0.38	46
Total water (0.1 kg)	17.98 ± 2.51	0.13	16.77 ± 2.83	0.02	1.56	0.13	46
Age of sample (years)	6.58 ± 0.35	0.41	6.46 ± 0.32	0.43	0.83	0.34	46

Legend: SD—standard deviation; SWp—level of statistical significance of Shapiro–Wilk coefficient; *t*—*t*-test values; *p*—level of statistical significance of *t*-test; df—freedom degrees.

**Table 2:** Descriptive statistics of KTK test variables—raw data.

Variables	Boys (N = 23)		Girls (N = 25)				
	Mean ± SD	SWp	Mean ± SD	SWp	*t*	*p*	df
balance beam (freq.)	20.70 ± 9.57	0.52	23.27 ± 8.99	0.53	−1.13	0.27	46
single-leg jumps (points)	7.48 ± 3.06	0.35	10.68 ± 4.63	0.00	−2.80	0.01	46
lateral jumps (freq.)	31.65 ± 9.50	0.01	30.36 ± 10.84	0.32	0.44	0.66	46
platform movement (freq.)	26.09 ± 5.43	0.61	24.80 ± 4.22	0.40	0.92	0.37	46
KTK total score (points)	85.91 ± 20.30	0.74	89.56 ± 22.42	0.01	−0.59	0.56	46

Legend: SD—standard deviation; SWp—level of statistical significance of Shapiro–Wilk coefficient; *t*—*t*-test values; *p*—level of statistical significance of *t*-test; df—freedom degrees.

**Table 3:** The results of regressive analysis for balance beam.

Variable	Boys				Girls			
	r	*p*	Beta	pbeta	r	*p*	Beta	pbeta
Body height	−0.11	0.30	0.44	0.58	−0.08	0.35	−0.16	0.76
Body mass	−0.17	0.22	−0.22	0.69	−0.08	0.36	−1.00	0.45
Total musle mass	−0.23	0.14	−0.50	0.85	−0.06	0.40	0.52	0.61
Total fat	−0.10	0.32	0.15	0.75	−0.03	0.44	0.50	0.52
Total water	−0.21	0.17	−0.06	0.99	−0.01	0.47	0.16	0.70
R	0.32				0.20			
R^2^	0.10				0.04			
P	0.86				0.97			

Legend: r—Pearson correlation coefficient; *p*—level of statistical significance for r; Beta—regressive coefficient; pbeta—level of regressive coefficient significance; R—multiple correlation coefficient; R^2^—determination coefficient; P—significance multiple correlation coefficient.

**Table 4:** Regressive analysis of single-leg jumps variable.

Variable	Boys				Girls			
	r	*p*	Beta	pbeta	r	*p*	Beta	pbeta
Body height	−0.23	0.14	−1.97	0.00	−0.05	0.40	−0.14	0.79
Body mass	0.16	0.23	−0.14	0.69	−0.02	0.46	−1.17	0.37
Total muscle mass	−0.13	0.28	−4.06	0.03	−0.01	0.48	0.46	0.65
Total fat	0.29	0.09	0.68	0.04	0.03	0.44	0.68	0.38
Total water	−0.10	0.33	5.36	0.02	0.05	0.40	0.28	0.48
R	0.79				0.25			
R^2^	0.62				0.06			
P	0.00				0.97			

Legend: r—Pearson correlation coefficient; *p*—level of statistical significance for r; Beta—regressive coefficient; pbeta—level of regressive coefficient significance; R—multiple correlation coefficient; R^2^—determination coefficient; P—significance multiple correlation coefficient.

**Table 5:** Regressive analysis of lateral jumps variable.

Variable	Boys				Girls			
	r	*p*	Beta	pbeta	r	*p*	Beta	pbeta
Body height	−0.34	0.06	−2.05	0.00	−0.34	0.06	−0.15	0.77
Body mass	−0.02	0.46	−0.31	0.44	−0.22	0.14	−0.18	0.88
Total muscle mass	−0.21	0.17	−4.30	0.03	−0.31	0.07	−0.20	0.84
Total fat	0.10	0.33	0.52	0.15	−0.09	0.33	0.31	0.67
Total water	−0.19	0.19	5.82	0.02	−0.29	0.08	−0.04	0.92
R	0.73				0.37			
R^2^	0.53				0.14			
P	0.02				0.70			

Legend: r—Pearson correlation coefficient; *p*—level of statistical significance for r; Beta—regressive coefficient; pbeta—level of regressive coefficient significance; R—multiple correlation coefficient; R^2^—determination coefficient; P—significance multiple correlation coefficient.

**Table 6:** Regressive analysis of platform movement variable.

Variable	Boys				Girls			
	r	*p*	Beta	pbeta	r	*p*	Beta	pbeta
Body height	0.06	0.40	−0.86	0.22	−0.09	0.34	0.14	0.77
Body mass	0.06	0.39	0.37	0.43	0.14	0.25	1.27	0.27
Total muscle mass	0.08	0.36	−4.18	0.08	−0.03	0.44	−0.86	0.34
Total fat	−0.15	0.25	−0.96	0.03	0.26	0.10	−0.10	0.89
Total water	0.10	0.31	5.43	0.07	−0.21	0.16	−0.49	0.18
R	0.56				0.52			
R^2^	0.32				0.28			
P	0.22				0.26			

Legend: r—Pearson correlation coefficient; *p*—level of statistical significance for r; Beta—regressive coefficient; pbeta—level of regressive coefficient significance; R—multiple correlation coefficient; R^2^—determination coefficient; P—significance multiple correlation coefficient.

**Table 7:** Regressive analysis of KTK overall score variable.

Variable	Boys				Girls			
	r	*p*	Beta	pbeta	r	*p*	Beta	pbeta
Body height	−0.23	0.15	−1.28	0.09	−0.22	0.14	−0.14	0.79
Body mass	−0.05	0.41	−0.17	0.74	−0.12	0.29	−0.49	0.70
Total muscle mass	−0.21	0.17	−3.98	0.11	−0.18	0.19	0.05	0.96
Total fat	0.01	0.50	0.16	0.37	0.01	0.50	0.47	0.54
Total water	−0.18	0.21	4.96	0.11	−0.17	0.21	0.01	0.98
R	0.49				0.28			
R^2^	0.26				0.08			
P	0.42				0.90			

Legend: r—Pearson correlation coefficient; *p*—level of statistical significance for r; Beta—regressive coefficient; pbeta—level of regressive coefficient significance; R—multiple correlation coefficient; R^2^—determination coefficient; P—significance multiple correlation coefficient.

## Data Availability

Data presented in this study are available upon request from the corresponding author.
